# Retinal and choroidal microvascular analysis by swept-source optical coherence tomography angiography in thyroid-associated ophthalmopathy (TAO) and hyperthyroidism without clinical signs of TAO

**DOI:** 10.1080/07853890.2025.2478314

**Published:** 2025-03-20

**Authors:** Congbi Liang, Jingcheng Liu, Dihao Hua, Tianyue Cao, Yang Meng, Di Xiao, Hongmei Zheng, Zhen Chen, Changzheng Chen, Yishuang Xu

**Affiliations:** a Eye Center, Renmin Hospital of Wuhan University, Wuhan, Hubei, China; b Department of Endocrinology & Metabolism, Renmin Hospital of Wuhan University, Wuhan, Hubei, China

**Keywords:** Thyroid-associated ophthalmopathy, hyperthyroidism, swept-source optical coherence tomography angiography, retinal vessel density, choroid thickness

## Abstract

**Objective:**

To analyse retinal and choroidal microvascular alterations in patients with thyroid-associated ophthalmopathy (TAO) and hyperthyroidism without signs of TAO using swept-source optical coherence tomography angiography (SS-OCTA) and to investigate the potential reasons for ocular microvascular changes in patients with TAO.

**Methods:**

Thirty patients with active TAO (group A), thirty patients with hyperthyroidism without signs of TAO (group B), and thirty healthy subjects (group C) were recruited. The images of macula and optic nerve head were obtained by SS-OCTA. Vessel density (VD) of superficial capillary plexus (SCP), deep capillary plexus, radial peripapillary capillary and choriocapillaris (CC), choroidal vascularity index, foveal avascular zone area, macular thickness, macular ganglion cell complex thickness, choroidal thickness (CT), and retinal nerve fibre layer thickness were measured.

**Results:**

Group A had significantly higher VD of SCP than group C (all *p* < 0.05), and group B had significantly lower VD of SCP than group C (all *p* < 0.05). The VD of CC was significantly lower in group A than in groups B and C (*p* < 0.05). CT was significantly thicker in groups A and B than in group C (all *p* < 0.05). Multivariable regression analysis showed that the free triiodothyronine was significantly negatively correlated with the VD of SCP in the parafoveal regions in groups B and C (*p* < 0.05) and proptosis was significantly positively correlated with the VD of SCP in groups A and B (all *p* < 0.05).

**Conclusion:**

We noted a difference in the VD of SCP and CC between patients with TAO and patients with hyperthyroidism without signs of TAO. These relative differences in ocular microvasculature indicated that local (orbital) and systemic (thyroid-related) factors may both play a role in disease presentation and progression, with orbital factors having a greater impact.

## 1. Introduction

Thyroid-associated ophthalmopathy (TAO) is an autoimmune disease characterized by the immune-mediated destruction of ocular tissues [1]. The pathologic changes commonly observed in TAO include extraocular muscle enlargement and orbital connective tissue hyperplasia. These develop secondary to increased inflammation, orbital fibroblast hyperactivation and differentiation, and hyaluronic acid over production [2,3]. These are responsible for the clinical manifestations in TAO, which include eyelid retraction, exophthalmos, diplopia, and visual impairment [4,5]. TAO also occasionally presented with vaso-occlusive conditions, such as retinal vein occlusion and glaucoma [6–8]. An increasing number of recent studies have brought attention to the role of optical coherence tomography angiography (OCTA) in detecting the ocular microvascular changes [9–13].

OCTA is a non-invasive diagnostic tool that can image the microvasculature of the retina and choroid [14,15]. Compared to the traditional OCTA, swept-source OCTA (SS-OCTA) boasts better image resolution and faster scanning speeds, because the latter uses light with longer wavelengths to penetrate the retinal pigment epithelium (RPE) more effectively. As such, SS-OCTA systems can scan past the RPE and into the choroid [16,17]. We theorize that the SS-OCTA is more appropriate for exploring the ocular microvascular changes associated with TAO, particularly those occurring in the choroidal layer.

A previous study utilized the OCTA to demonstrate increased retinal microvascular density in active TAO [10]. However, another study published contrary findings, stating that retinal microvascular density was actually significantly lower in patients with active TAO group than in healthy subjects [9]. As such, the retinal microvascular changes associated with TAO remain controversial. Existing literature proposes that the ocular microvascular changes in TAO develop secondary to thyroid-associated inflammatory mediators that contribute to orbital swelling and inflammation [9,10,18]. However, there is limited data on these circulating factors, and their role in such conditions remains unclear. A better understanding of TAO pathogenesis is important, because it can shed light on the clinical course of the disease. Moreover, only a few studies have explored the ocular microvascular changes associated with hyperthyroidism. This study utilized the SS-OCTA to identify the retinal and choroidal microvascular changes in patients with TAO and patients with hyperthyroidism without signs of TAO and proposed possible reasons for such.

## 2. Methods

### 2.1. Participants

This was an observational, cross-sectional study which was conducted from June 2022 to October 2022 at Renmin Hospital of Wuhan University. Active TAO patients combined with hyperthyroidism (group A), hyperthyroidism patients without signs of TAO (group B), and healthy subjects (group C) were recruited. Approval for this study was granted by the Renmin Hospital of Wuhan University, and all research and data collection complied with the Declaration of Helsinki. Written informed consent was obtained from each subject before study.

The inclusion criteria for TAO patients were as follows: (1) meeting the Bartley diagnostic criteria [19], (2) combined with hyperthyroidism, (3) clinical activity score ≥ 3, and (4) without treatment such as glucocorticoid therapy, radiation therapy, or surgery before the study. The inclusion criteria for hyperthyroidism patients were as follows: (1) meeting the diagnostic criteria for hyperthyroidism as set by the American Thyroid Society in 2016 [20], and (2) without any ocular lesions. The exclusion criteria for all patients were as follows: (1) age < 18 years, (2) refractive error > ± 3 diopters, (3) best-corrected visual acuity < 20/30, (4) any history of ocular diseases (cataract, glaucoma, retinal disease, dysthyroid optic neuropathy or any optic neuropathy) or surgeries, (5) systemic diseases that could affect retinal microcirculation, and (6) smoking.

Clinical data of subjects were collected, including heart rate, blood pressure (BP), free triiodothyronine (FT3), free thyroxine (FT4), and thyroid stimulating hormones (TSH). All participants underwent a detailed ophthalmic examination, including slit-lamp biomicroscopy, best-corrected visual acuity, intraocular pressure (IOP) measurements with a noncontact tonometer, proptosis, and SS-OCTA. Proptosis was measured by the same examiner who was experienced in Hertel exophthalmometry. Randomly select one eye of participants for inclusion in the study.

### 2.2. SS-OCTA imaging

The SS-OCTA (VG100; SVision Imaging, Ltd., Luoyang, China) system contained an SS laser with a central wavelength of approximately 1050 nm and a scan rate of 100,000 A-scans per second. The maximum axial and estimated lateral resolutions in tissue were approximately 5 µm and 15 µm, respectively. The scan depth was 3 mm.

**Figure 1.**
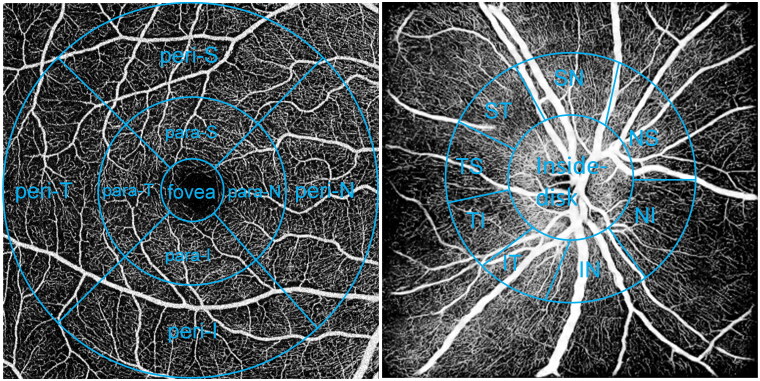
Nine measurement zones of the macula (left) and optic disc (right). Para: parafoveal; peri: perifoveal; I: inferior; T: temporal; S: superior; N: nasal; NS: nasal superior; NI: nasal inferior; IN: inferior nasal; IT: inferior tempo; TI: tempo inferior; TS: tempo superior; ST: superior tempo; SN: superior nasal.

**Figure 2.**
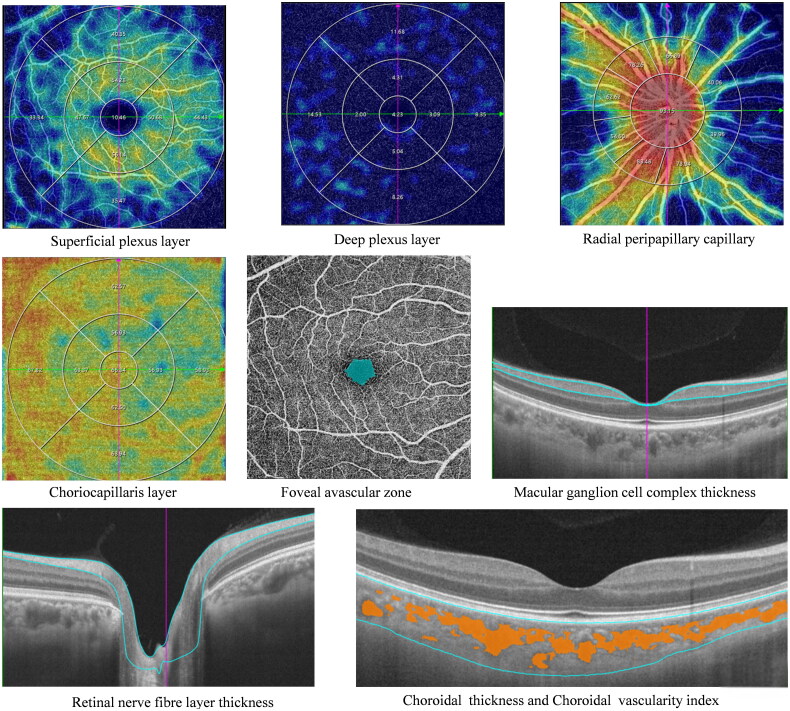
The measurements of retinal and choroidal parameters of a TAO patient. All parameters are measured automatically by built-in software.

All participants underwent SS-OCTA with the imaging model of macular angio 6 × 6 mm 512 × 512 R4 scans and ONH angio 6 × 6 mm 512 × 512 R4 scans. The macular subfields consisted of three concentric circle images with diameters of 1 mm (foveal area), 1–3mm (parafoveal area), and 3–6mm (perifoveal area). The parafoveal and perifoveal area were respectively subdivided into four parts: superior (S), inferior (I), nasal (N), and temporal (T) (Figure 1). The measurement area around the optic disc was divided into nine subfields: inside-disc, nasal inferior (NI), inferior nasal (IN), inferior tempo (IT), tempo inferior (TI), tempo superior (TS), superior tempo (ST), superior nasal (SN), and nasal superior (NS) (Figure 1). The vessel density (VD) of superficial capillary plexus (SCP), deep capillary plexus (DCP), radial peripapillary capillary (RPC) and choriocapillaris (CC), choroidal vascularity index (CVI), area of foveal avascular zone (FAZ), macular thickness (MT), macular ganglion cell complex (GCC) thickness, choroidal thickness (CT), and retinal nerve fibre layer (RNFL) thickness were measured by the software built in the equipment (Figure 2). Images with a scan quality better than 7/10 were included in the study. All OCTA images were captured by a trained ophthalmic photographer under consistent conditions.

### 2.3. Statistics

Statistical analysis was performed using the SPSS 25.0 program (SPSS Chicago, Illinois, USA). The data are presented as the means ± standard deviation. The Kolmogorov–Smirnov test was performed to check the normality of the data distributions. For normal-distributed data, one-way ANOVA test was used for data comparisons among the three groups, and the least square difference test was subsequently performed for group comparisons when homogeneity of variance was met. Otherwise, a Tamhane’s T2 test was used for group comparisons. For non-normal-distributed data, we used the Kruskal-Wallis *H* test. Multivariable regression analysis was performed to assess the combined effects of thyroid hormone levels, heart rate, BP, and proptosis on the VD of SCP. Data were considered significant at *p* < 0.05.

## 3. Results

### 3.1. Demographics and clinical characteristics

Ninety eyes from ninety participants were enrolled in this study. Thirty eyes (6 males and 24 females) were assigned to the active TAO patients with hyperthyroidism group. Thirty eyes (9 males and 21 females) were assigned to the hyperthyroidism patients without signs of TAO group. The last thirty eyes (8 males and 22 females) were assigned to the group of healthy subjects. There was no difference in sex distribution, age, IOP, and BP among the three groups (all *p* > 0.05). As expected, proptosis was significantly higher in group A than in groups B and C (group A vs. group B, *p* < 0.001; group A vs. group C, *p* < 0.001). Heart rate was higher in groups A and B than in group C (group A vs. group C, *p* < 0.001; group B vs. group C, *p* < 0.001). Free thyroid hormone (FT3 and FT4) and TSH were higher and lower, respectively, in groups A and B than in group C (group A vs. group C, *p* < 0.001; group B vs. group C, *p* < 0.001) (Table 1).

**Table 1.** Demographic and clinical data of all enrolled subjects.

	Group A (*n* = 30)	Group B (*n* = 30)	Group C (*n* = 30)	*p-*value
Age (years)	45.07 ± 2.69	44.63 ± 3.18	44.00 ± 2.98	0.451*
IOP (mmHg)	14.86 ± 1.69	15.07 ± 1.56	14.86 ± 1.97	0.837*
Proptosis (mm)	16.52 ± 1.20^a^^b^	12.95 ± 0.63	12.84 ± 0.66	<0.001*
Heart rate (beats/min)	100.27 ± 5.17^a^	102.10 ± 5.54^a^	84.67 ± 4.77	<0.001*
Systolic blood pressure (mmHg)	120.80 ± 7.53	120.77 ± 8.16	118.80 ± 7.46	0.344*
Diastolic blood pressure (mmHg)	78.97 ± 3.83	79.63 ± 3.55	78.17 ± 3.73	0.242*
FT3 (pg/ml)	6.62 ± 1.62^a^	6.72 ± 1.52^a^	3.19 ± 0.75	<0.001*
FT4 (ng/dl)	3.31 ± 1.02^a^	3.49 ± 1.00^a^	1.25 ± 0.19	<0.001*
TSH (μIU/ml)	0.11 ± 0.99^a^	0.13 ± 0.10^a^	2.15 ± 0.94	<0.001*

Data are the means ± standard deviations.

^*^ Kruskal-Wallis *H* test.

^a^ statistically significant results (*p* < 0.05) compare with Group C.

^b^statistically significant results (*p* < 0.05) compare with Group B.

IOP, intraocular pressure; FT3, free triiodothyronine; FT4, free thyroxine; TSH, thyroid stimulating hormones.

### 3.2. VD of retina and choroid

**Figure 3.**
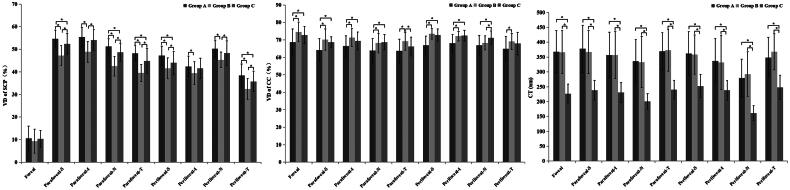
Bars show the vessel density of superficial capillary plexus (left) and choriocapillaris (middle), and choroidal thickness (right) in nine regions in TAO patients (group A), hyperthyroidism patients (group B), and healthy controls (group C) (mean ± standard error of the mean). *: statistically significant results (*p* < 0.05).

The VD of SCP, DCP, RPC, and CC analysis results are summarized in Tables 2 and 3. There were significant differences among the three groups with regards to the VD of SCP (*p* < 0.05, for most areas). In the *post hoc* pairwise analysis, group A had significantly higher VD of SCP (*p* < 0.05) compared to group C, except in the parafoveal-I, perifoveal-I, and perifoveal-N regions. Comparatively, group B had significantly lower VD of SCP (*p* < 0.05) compared to group C, except in the perifoveal-I region (Figure 3). There were no significant differences in the VD of DCP and RPC were not significant among the three groups (all *p* > 0.05), but there was a significant differences in the VD of CC among the three groups (*p* < 0.05). In the *post hoc* pairwise analysis, group A had significantly lower VD of CC values than group C (*p* < 0.05), except in the parafoveal-T and perifoveal-T regions (Figure 3). There were no significant differences in the VD of CC between groups B and C (*p* > 0.05), except in the parafoveal-T and perifoveal-N regions.

**Table 2.** Vessel density of superficial capillary plexus and deep capillary plexus analysis results in the three groups.

Characteristic	Group A (*n* = 30)	Group B (*n* = 30)	Group C (*n* = 30)	*p-*value
Vessel density of superficial capillary plexus (%)				
Foveal	10.59 ± 5.40	9.33 ± 5.26	10.31 ± 3.77	0.174
Parafoveal-S	54.62 ± 3.87^ab^	47.26 ± 4.31^a^	52.42 ± 4.54	<0.001*
Parafoveal-I	55.42 ± 4.34^b^	48.85 ± 4.53^a^	54.09 ± 4.60	<0.001*
Parafoveal-N	51.28 ± 3.37^ab^	42.54 ± 4.38^a^	48.65 ± 3.87	<0.001*
Parafoveal-T	48.19 ± 3.38^ab^	39.52 ± 3.76^a^	44.84 ± 5.66	<0.001*
Perifoveal-S	47.15 ± 4.27^ab^	41.56 ± 4.59^a^	44.00 ± 5.06	<0.001*
Perifoveal-I	42.40 ± 4.58^b^	39.46 ± 5.09	41.52 ± 4.60	0.054*
Perifoveal-N	50.23 ± 3.85^b^	45.33 ± 3.47^a^	48.32 ± 5.41	<0.001*
Perifoveal-T	38.44 ± 5.01^ab^	32.43 ± 4.65^a^	35.71 ± 4.57	<0.001*
Vessel density of deep capillary plexus (%)				
Foveal	2.39 ± 2.58	2.65 ± 3.00	3.80 ± 3.76	0.210
Parafoveal-S	6.63 ± 3.45	8.38 ± 4.86	7.25 ± 4.71	0.300*
Parafoveal-I	8.49 ± 4.43	8.64 ± 6.32	8.94 ± 5.58	0.834
Parafoveal-N	6.88 ± 4.47	7.73 ± 5.52	8.11 ± 4.65	0.386
Parafoveal-T	8.93 ± 4.20	9.01 ± 5.80	10.39 ± 5.21	0.333
Perifoveal-S	12.72 ± 4.66	13.10 ± 5.67	13.87 ± 5.64	0.699*
Perifoveal-I	14.05 ± 5.17	12.80 ± 5.74	14.20 ± 6.55	0.502
Perifoveal-N	9.30 ± 5.26	8.31 ± 7.24	9.40 ± 6.90	0.329
Perifoveal-T	21.05 ± 6.28	18.84 ± 6.87	22.04 ± 7.20	0.177

Data are the means ± standard deviations.

S, superior; I, inferior; N, nasal; T, temporal.

^*^ one-way ANOVA test. Others: Kruskal-Wallis *H* test.

^a^ statistically significant results (*p* < 0.05) compare with Group C.

^b^ statistically significant results (*p* < 0.05) compare with Group B.

**Table 3.** Vessel density of radial peripapillary capillary and choriocapillaris analysis results in the three groups.

	Group A (*n* = 30)	Group B (*n* = 30)	Group C (*n* = 30)	*p-*value
Vessel density of radial peripapillary capillary (%)				
Inside-disc	92.87 ± 2.63^a^	92.10 ± 3.05	91.00 ± 2.16	0.025*
NI	43.27 ± 12.77	44.42 ± 9.53	40.86 ± 9.87	0.434*
IN	70.37 ± 10.81	66.66 ± 8.62	68.30 ± 7.54	0.216
IT	82.62 ± 4.40	81.65 ± 4.58	80.92 ± 4.95	0.370*
TI	56.31 ± 11.42	59.38 ± 9.55	53.39 ± 7.98	0.071
TS	58.03 ± 10.59	54.47 ± 8.96	54.68 ± 7.38	0.380
ST	79.93 ± 4.92	78.50 ± 4.73	77.74 ± 4.41	0.191*
SN	70.76 ± 7.98	72.91 ± 5.39	68.75 ± 7.31	0.075*
NS	55.11 ± 9.03	54.69 ± 8.55	53.29 ± 7.03	0.672*
Vessel density of choriocapillaris (%)				
Foveal	68.73 ± 7.59^a^^b^	74.59 ± 5.57	72.79 ± 5.00	0.001*
Parafoveal-S	64.14 ± 6.63^a^^b^	70.19 ± 6.03	68.64 ± 3.18	<0.001*
Parafoveal-I	66.53 ± 5.85^a^^b^	71.38 ± 5.22	69.44 ± 5.09	0.003*
Parafoveal-N	63.86 ± 7.06^a^^b^	68.19 ± 5.66	68.73 ± 4.30	0.003*
Parafoveal-T	63.81 ± 6.65^b^	69.28 ± 5.09^a^	66.20 ± 5.40	0.002*
Perifoveal-S	66.90 ± 5.36^a^^b^	73.46 ± 3.04	72.71 ± 3.50	<0.001*
Perifoveal-I	68.08 ± 5.01^a^^b^	72.14 ± 2.72	72.47 ± 3.06	<0.001*
Perifoveal-N	66.93 ± 5.65^a^^b^	68.30 ± 3.97^a^	71.18 ± 3.87	0.002*
Perifoveal-T	64.88 ± 7.23^b^	69.10 ± 4.58	67.92 ± 6.44	0.028*

Data are the means ± standard deviations.

NS: nasal superior; NI: nasal inferior; IN: inferior nasal; IT: inferior tempo; TI: tempo inferior; TS: tempo superior; ST: superior tempo; SN: superior nasal; S: superior; I: inferior; N: nasal; T: temporal.

^*^ one-way ANOVA test; Others: Kruskal-Wallis *H* test.

^a^ statistically significant results (*p* < 0.05) compare with Group C.

^b^ statistically significant results (*p* < 0.05) compare with Group B.

### 3.3. CT and CVI

The CT and CVI analysis results are listed in Table 4. Statistically significant differences were found among the three groups for all regions of CT (all *p* < 0.05). In the *post hoc* pairwise analysis, the CT of all regions in groups A and B were significantly thicker than in group C (all *p* < 0.05) (Figure 3). There was no significant difference in CVI of any regions among the three groups (all *p* > 0.05).

**Table 4.** Choroidal thickness and choroidal vascularity index analysis results in the three groups.

Characteristic	Group A (*n* = 30)	Group B (*n* = 30)	Group C (*n* = 30)	*p-*value
Choroidal thickness (μm)				
Foveal	368.07 ± 70.63^a^	366.40 ± 72.17^a^	226.73 ± 32.12	<0.001
Parafoveal-S	377.87 ± 79.38^a^	366.93 ± 72.39^a^	238.80 ± 33.19	<0.001*
Parafoveal-I	356.50 ± 77.19^a^	356.93 ± 78.95^a^	230.47 ± 33.94	<0.001*
Parafoveal-N	336.03 ± 72.46^a^	332.77 ± 86.03^a^	200.40 ± 25.72	<0.001*
Parafoveal-T	369.90 ± 62.14^a^	372.80 ± 69.99^a^	240.27 ± 32.00	<0.001*
Perifoveal-S	361.53 ± 74.22^a^	358.37 ± 66.34^a^	252.40 ± 38.87	<0.001*
Perifoveal-I	336.77 ± 75.47^a^	331.87 ± 90.78^a^	238.23 ± 33.19	<0.001*
Perifoveal-N	279.77 ± 63.59^a^	292.83 ± 75.17^a^	161.13 ± 25.13	<0.001
Perifoveal-T	348.37 ± 68.23^a^	367.90 ± 60.95^a^	247.67 ± 41.26	<0.001
Choroidal vascularity index				
Foveal	0.39 ± 0.08	0.37 ± 0.08	0.37 ± 0.10	0.577
Parafoveal-S	0.36 ± 0.07	0.35 ± 0.08	0.34 ± 0.09	0.426
Parafoveal-I	0.38 ± 0.08	0.38 ± 0.08	0.36 ± 0.09	0.703*
Parafoveal-N	0.39 ± 0.07	0.37 ± 0.08	0.37 ± 0.10	0.640*
Parafoveal-T	0.37 ± 0.07	0.36 ± 0.08	0.37 ± 0.08	0.926*
Perifoveal-S	0.37 ± 0.06	0.35 ± 0.06	0.34 ± 0.06	0.210*
Perifoveal-I	0.38 ± 0.07	0.37 ± 0.07	0.36 ± 0.07	0.374*
Perifoveal-N	0.38 ± 0.06	0.38 ± 0.07	0.35 ± 0.09	0.278
Perifoveal-T	0.34 ± 0.05	0.34 ± 0.07	0.33 ± 0.06	0.856

Data are the means ± standard deviations.

S, superior; I, inferior; N, nasal; T, temporal.

^*^ one-way ANOVA test; Others: Kruskal-Wallis *H* test.

^a^ statistically significant results (*p* < 0.05) compare with Group C.

### 3.4. RNFL and GCC thickness, MT, and FAZ area

RNFL and GCC thickness, MT, and FAZ area analysis results are listed in Table 5. There were no significant differences in RNFL and GCC thickness, MT, and FAZ area among the three groups (*p* = 0.801, *p* = 0.224, *p* = 0.914, *p* = 0.463, respectively).

**Table 5.** Foveal avascular zone area, macular thickness, retinal nerve fibre layer thickness, and ganglion cell complex thickness analysis results in the three groups.

	Group A (*n* = 30)	Group B (*n* = 30)	Group C (*n* = 30)	*p-*value
Macular thickness (μm)	206.11 ± 11.79	206.73 ± 15.15	205.24 ± 13.81	0.914^*^
RNFL thickness (μm)	109.33 ± 9.92	107.70 ± 7.93	107.24 ± 12.82	0.801^*^
GCC thickness (μm)	81.94 ± 3.54	80.94 ± 4.15	82.71 ± 4.16	0.224^*^
Area of FAZ (mm^2^)	0.42 ± 0.16	0.44 ± 0.15	0.40 ± 0.08	0.463^*^

Data are the means ± standard deviations.

RNFL, retinal nerve fibre layer; GCC, ganglion cell complex; FAZ, foveal avascular zone.

^*^ one-way ANOVA test.

### 3.5. Correlation analysis of thyroid hormone levels, heart rate, BP, and proptosis with the VD of SCP

The multivariable regression analysis results between thyroid hormone levels, heart rate, BP, and proptosis with the VD of SCP are listed in Tables 6 and 7. Our results showed a significant negative correlation between FT3 and the VD of SCP in the parafoveal regions in groups B and C (*p* < 0.05). Our results also showed a significant positive correlation between proptosis and the VD of SCP in groups A and B (all *p* < 0.05).

**Table 6.** Multivariable regression analysis to identify factors associated with vessel density of superficial capillary plexus in groups B and C.

	FT3		TSH		Systolic BP		Diastolic BP		Heart rate		Proptosis	
	β	*p*	β	*p*	β	*p*	β	*p*	β	*p*	β	*p*
Foveal	−0.399	0.073	0.070	0.760	−0.080	0.605	0.093	0.504	0.267	0.306	−0.088	0.510
Parafoveal-S	−0.802	<0.001	0.131	0.404	−0.102	0.337	−0.060	0.526	0.277	0.121	−0.128	0.165
Parafoveal-I	−0.570	0.002	−0.083	0.647	−0.049	0.668	−0.066	0.550	−0.102	0.618	−0.174	0.103
Parafoveal-N	−0.807	<0.001	−0.054	0.705	−0.114	0.245	−0.022	0.803	0.070	0.664	−0.208	0.016
Parafoveal-T	−0.551	0.003	0.238	0.201	−0.108	0.389	−0.030	0.791	0.209	0.319	−0.147	0.177
Perifoveal-S	−0.127	0.562	0.120	0.600	0.153	0.324	−0.131	0.344	−0.078	0.762	−0.024	0.856
Perifoveal-I	0.123	0.584	0.064	0.785	0.161	0.313	−0.014	0.920	−0.326	0.220	0.006	0.964
Perifoveal-N	−0.159	0.458	0.188	0.401	0.099	0.515	0.004	0.974	−0.090	0.721	−0.005	0.972
Perifoveal-T	0.086	0.683	0.255	0.251	0.240	0.114	−0.102	0.447	−0.201	0.421	−0.139	0.281

FT3, free triiodothyronine; TSH, thyroid stimulating hormones; BP, blood pressure; S, superior; I, inferior; N, nasal; T, temporal.

**Table 7.** Multivariable regression analysis to identify factors associated with vessel density of superficial capillary plexus in groups A and B.

	FT3		TSH		Systolic BP		Diastolic BP		Heart rate		Proptosis	
	β	*p*	β	*p*	β	*p*	β	*p*	β	*p*	β	*p*
Foveal	−0.177	0.228	0.008	0.954	0.007	0.964	0.023	0.899	−0.031	0.835	0.118	0.393
Parafoveal-S	−0.271	0.013	−0.096	0.367	0.010	0.931	−0.026	0.794	−0.048	0.656	0.631	<0.001
Parafoveal-I	−0.122	0.314	−0.043	0.723	−0.021	0.868	0.028	0.808	−0.032	0.795	0.569	<0.001
Parafoveal-N	−0.232	0.024	−0.096	0.341	−0.043	0.676	0.015	0.876	−0.020	0.842	0.698	<0.001
Parafoveal-T	−0.141	0.161	−0.087	0.384	−0.021	0.839	0.021	0.226	−0.080	0.428	0.715	<0.001
Perifoveal-S	0.037	0.769	−0.043	0.732	0.098	0.454	0.009	0.940	0.137	0.289	0.512	<0.001
Perifoveal-I	0.257	0.063	0.064	0.638	0.131	0.353	0.070	0.591	−0.026	0.850	0.285	0.031
Perifoveal-N	0.002	0.988	−0.018	0.886	0.065	0.608	0.083	0.476	0.183	0.148	0.550	<0.001
Perifoveal-T	0.217	0.082	0.020	0.874	0.023	0.854	0.050	0.668	0.187	0.141	0.514	<0.001

FT3, free triiodothyronine; TSH, thyroid stimulating hormones; BP, blood pressure; S, superior; I, inferior; N, nasal; T, temporal.

## 4. Discussion

TAO was an autoimmune disease and sometimes followed the onset of hyperthyroidism. The common changes in TAO were extraocular muscle thickening and orbital adipose tissue hyperplasia, which could compress the orbit and affect ocular blood flow. As such, this study examined retinal and choroidal microvascular changes in patients with active TAO and patients with hyperthyroidism without TAO using the SS-OCTA.

Our study found lower VD of SCP in group B compared to group C. A significant negative correlation between FT3 and the VD of SCP in the parafoveal regions was also observed. This change was speculated to be caused by an increase in plasma endothelin-1, which was found to be positively correlated with FT3 levels in patients with hyperthyroidism [21,22]. Endothelin-1 is a potent vasoconstrictor that is produced by retinal endothelial cells. Endothelin-1 receptors are distributed in the choroid and retinal arteries, making it a significant determinant of the reducing of macular blood flow [22]. The deposition of thyroid-related antibodies in the ocular microvascular system may also induce the inflammatory process, resulting in the loss of capillary density [23]. The VD of SCP may be a potential indicator for monitoring hyperthyroidism. This study identified alterations in the VD of SCP in group B, and also found that compared to group C, group A had higher VD of SCP. This indicated that retinal microvascular alterations might involve in the mechanism of both TAO and hyperthyroidism development. While the specific relationship between retinal microcirculatory changes and disease progression remains unclear, several factors were speculated to impact the vascular changes in previous studies [10,18]. Ye et al. [10] demonstrated increased retinal superficial microvascular density in patients with active TAO, which may have developed secondary to the increased orbital blood flow from extraocular muscle enlargement and orbital inflammation [10,24]. In comparison, Jamshidian et al. [18] found a significant reduction in the VD of SCP in patients with active TAO. This change may be due to vascular changes secondary to orbital tissue swelling and elevated plasma endothelin-1 levels. Although the impact of different devices used to obtain VD and individual differences between included objects might affect the final results in various studies, different mechanisms could all contribute to the changes in retinal VD in patients with TAO and showed different influence intensity in various TAO individuals. In our study, the results demonstrated a significant positive correlation between the VD of SCP and proptosis in groups A and B, while thyroid hormone levels, blood pressure, and heart rate were not significantly correlated with the VD of SCP. As such, we theorize that increased VD of SCP in patients of TAO with hyperthyroidism may primarily be due to orbital microvascular dilatation secondary to extraocular muscle enlargement and orbital inflammation [10,24]. Future research may focus on this correlation to better elucidate the pathogenesis behind visual impairment in TAO and other related disorders.

The VD of CC, CT, and CVI could be used as indices to assess choroidal structure and blood flow. Our study found that the CT in group A was significantly higher than in group C, which was consistent with previous research [25,26]. The increase in the CT of patients with TAO may be related to venous outflow obstruction secondary to reduced choroidal drainage and ophthalmic vein congestion [27]. It may also be related to choroidal stromal thickening caused by choroidal stromal fibroblast activation and increased glycosaminoglycan synthesis [28]. Our study also showed significantly higher CT values in group B compared to group C, which indicates that structural choroidal changes may occur prior to the development of TAO. Hyperthyroidism is characterized by a hyperdynamic cardiovascular state that could increase cardiac output, as well as choroidal perfusion [29]. Future research should examine the possible mechanisms for this phenomenon. Moreover, there was no significant difference in the CVI among the three groups in this study, which coincides with existing literature [28]. It is theorize that while CT increases in patients with TAO, the structural changes in the stroma and vasculature may be proportional. As such, CVI is not significantly affected [28]. Our study also found no significant difference in the VD of CC in most of the regions in groups B and C. Comparatively, the VD of CC in group A was significantly lower compared to group C, which was also consistent with previous studies [13,30]. FT3 and FT4 levels were significantly higher in groups A and B compared to group C. Elevated thyroid hormone levels may increase heart rate, which can contribute to a hyperdynamic cardiovascular state that also increases orbital blood flow [29]. However, our study found no significant increase in the CC VD of patients with hyperthyroidism without TAO. This may be due to the sympathetic control of choroidal blood flow, which prevents excessive ocular perfusion through vasoconstriction [31]. Our study also identified a significant decrease in the CC VD of patients with active TAO. This decrease may be due to vascular compression from extraocular muscle enlargement and adipose tissue proliferation [30]. It is important to note that some patients with active TAO had no systemic symptoms. Further exploration of these patients might help to better analyze systemic factors’ impact on the microvascular changes of TAO.

This study also found no significant difference in the VD of DCP among the three groups, which was consistent with previous studies in TAO patients [11,32]. Similar phenomenon was also found in hypertension patients, and the mechanism was speculated as an increased autoregulatory capacity of DCP [33,34]. No significant difference was also noted in the FAZ area, MT and macular GCC thickness among the three groups, which consistent with previous studies [18,35]. All three groups also showed similar VD of RPC and RNFL thickness, which was consistent with a previous study that examined patients with active and inactive TAO patients without dysthyroid optic neuropathy [36].

Our preliminary study has several limitations. First, we followed a cross-sectional study design, which limited the generalizability of our data. Second, we included a small number of patients in this study. Future research can benefit from a larger sample population. We also recommend stratifying patients with inactive TAO, active TAO, and active TAO with dysthyroid optic neuropathy.

In conclusion, our study showed that patients with TAO had higher VD of SCP, lower VD of CC and thicker CT, whereas patients with hyperthyroidism without signs of TAO had lower VD of SCP, thicker CT, and no significant changes in CC. Our results demonstrated that there were differences in ocular microcirculation in the setting of TAO and hyperthyroidism without TAO. These relative differences in the identified factors indicate that the factors of orbital and hyperthyroidism have different effects on ocular microcirculation. Overall, orbital factors seem to influence these presentation most significantly.

## Data Availability

The data that support the findings of this study are available from the corresponding author, Professor Yishuang Xu, upon reasonable request.
